# Low Serum Total Testosterone Is Associated with Non-Alcoholic Fatty Liver Disease in Men but Not in Women with Type 2 Diabetes Mellitus

**DOI:** 10.1155/2022/8509204

**Published:** 2022-08-27

**Authors:** Xinxin Zhang, Jinfeng Xiao, Qi Liu, Yuanyuan Ye, Weihong Guo, Jingqiu Cui, Qing He, Wenli Feng, Ming Liu

**Affiliations:** Department of Endocrinology and Metabolism, Tianjin Medical University General Hospital, Tianjin, China

## Abstract

**Materials and Methods:**

There were 1155 patients with T2DM included in the analysis. Serum levels of total testosterone and the precursors of androgens, including androstenedione, DHEA, and DHEAS, were quantified using liquid chromatography-tandem mass spectrometry assays.

**Results:**

The risk of NAFLD decreased as total testosterone concentration increased in men with T2DM. After adjusting for age, current smoking, current drinking, body mass index, duration of T2DM, diastolic blood pressure, total cholesterol, triglycerides, low-density lipoprotein/high-density lipoprotein cholesterol ratio, uric acid, C-reactive protein, and sex hormones in model 4, the adjusted odds ratio (OR) and 95% confidence interval (CI) of NAFLD for tertile3 vs tertile1 was 0.37 (0.17–0.77; *P* = 0.024 for trend). When taken as a continuous variable, this association was still robust in model 4 (OR, 0.58; 95% CI, 0.42–0.80; *P* < 0.05). No significant associations were found between increasing levels of the precursors of androgens and the odds of NAFLD in men with T2DM (all *P* > 0.05). Moreover, women showed no significant associations of total testosterone, androstenedione, DHEA, and DHEAS, with the odds of NAFLD (all *P* > 0.05).

**Conclusions:**

Serum total testosterone was independently associated with the risk of NAFLD among men with T2DM. This study highlights the potential role of testosterone as a risk factor for NAFLD in patients with T2DM.

## 1. Introduction

Non-alcoholic fatty liver disease (NAFLD) is a metabolic liver disease, which has become a major health issue, affecting 25% of the adult population worldwide [1]. NAFLD ranges from simple fatty liver to non-alcoholic steatohepatitis (NASH) and may progress to liver cirrhosis and cancer [2]. Due to the increasingly unhealthy lifestyles in recent years, China has the highest prevalence and incidence of NAFLD in Asia [3]. Moreover, NAFLD with metabolic syndrome increases the risk of all-cause, liver-specific, and cardiovascular death [4].

Previous studies have reported that men and women differ in their susceptibility to metabolic diseases [5]. Accumulating evidence has demonstrated that sex steroid hormones play a significant role in the progression of cardiovascular and metabolic diseases. The sex and menopausal status differences in the NAFLD incidence [6–8] suggest the potential role of sex hormones as mediators in the progression of this disease. The prevalence of NAFLD is higher among patients with metabolic diseases than in the general population [9]; it is predicted that 75% of patients with type 2 diabetes mellitus (T2DM) have some form of NAFLD [10]. In these patients, it is necessary to identify the factors that are related to a high risk of NAFLD. Low testosterone is independently related to hepatic steatosis and NASH in men [11–13]. However, in patients with T2DM, few research studies have reported the association of testosterone and the precursors of androgens with risk of NAFLD, including androstenedione, dehydroepiandrosterone (DHEA), and dehydroepiandrosterone sulfate (DHEAS).

Accordingly, the aim of this cross-sectional study was to explore the associations of testosterone, androstenedione, DHEA, and DHEAS with the risk of NAFLD in patients with T2DM.

## 2. Materials and Methods

### 2.1. Study Subjects

**Figure 1:**
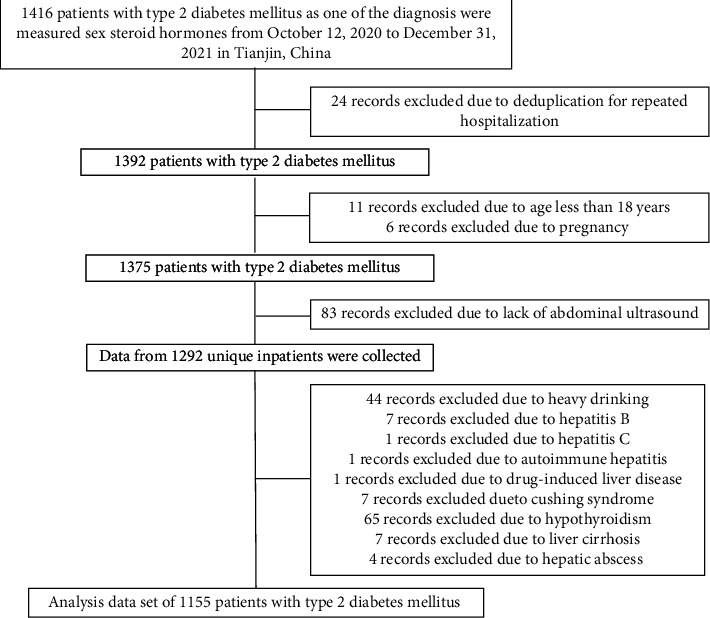
Flowchart of identification of study population. Based on the exclusion criteria, a total of 1155 patients with T2DM were included in the final analysis.

The medical records of 1,416 consecutive patients with T2DM were reviewed from October 12, 2020, to December 31, 2021. All patients were hospitalized, and their sex steroid hormone levels were measured at the Department of Endocrinology and Metabolism, Tianjin Medical University General Hospital. If multiple inpatient records were available for the same individual, only one record was selected. The exclusion criteria were as follows: (1) lack of abdominal ultrasonography examination; (2) ethanol consumption of ≥210 g/week for men and ≥140 g/week for women; and (3) viral or autoimmune hepatitis, drug-induced liver disease, Wilson's disease, Cushing's syndrome, and hypothyroidism.  Figure 1 illustrates the study population identification process. Based on the exclusion criteria, a total of 1155 patients with T2DM were included in the final analysis.

This study was approved by the institutional review board of Tianjin Medical University General Hospital. The requirement for informed consent was waived (approval number: IRB2020-YX-027-01) because the patient information was extracted from electronic medical records at the Department of Endocrinology and Metabolism, and the patients' identities were kept anonymous, except for the date of birth.

### 2.2. Information Collection and Measurements of Androgens

Data including age, sex, lifestyle characteristics, insurance, medical history of diabetes, hypertension and dyslipidemia, height, weight, systolic blood pressure, diastolic blood pressure, glycosylated hemoglobin (HbA1c), total cholesterol (TC), triglycerides (TG), high-density lipoprotein (HDL), low-density lipoprotein (LDL), uric acid (UA), C-reactive protein (CRP), aspartate aminotransferase (AST), and alanine aminotransferase (ALT) were obtained from the medical records. Body mass index (BMI) was calculated as the patients' weight in kilograms divided by the square of the height in meters. Alcohol drinking was assessed by self-report. Excess alcohol consumption was defined as drinking more than 210 g/week for men and 140 g/week for women, respectively.

Total testosterone (TT) and precursors of androgens, including androstenedione, DHEA, and DHEAS, were quantified using liquid chromatography-tandem mass spectrometry (LC-MS/MS) assays in the Laboratory of Endocrinology and Metabolism at Tianjin Medical University General Hospital. Patients' venous blood samples were drawn in the morning after hospital admission following at least 10 hours of fasting. An extraction liquid for steroids (Hangzhou Calibra Diagnostics Co., LTD., Zhejiang, China) was used for sample pretreatment. Androgens were measured using a Jasper™ HPLC system coupled to an AB SCIEX Triple Quad™ 4500MD mass spectrometer with a heated nebulizer ionization source in positive ion mode. Quantitative data analysis was performed using MultiQuant™ MD 3.0.2 software. Calibration curves of each set of samples were generated by plotting the area ratios of the analyte to the internal standard peak of the six calibration standards; two quality control samples were contained in each set of samples. The plot used linear regression with 1/*x*^2^ weighting, and the correlation coefficients were all greater than 0.99.

### 2.3. Definitions

Diabetes was defined as a fasting blood glucose level ≥7.0 mmol/L, 2-hour blood glucose level ≥11.1 mmol/L, HbA1c level ≥6.5%, previous medical history of diagnosed diabetes, or use of antidiabetic drugs [14]. Dyslipidemia was defined as TC ≥ 6.2 mmol/L, TG ≥ 2.3 mmol/L, LDL cholesterol ≥4.1 mmol/L, HDL cholesterol <1.0 mmol/L, or use of lipid lowering drugs [15]. Hypertension was defined as a systolic blood pressure ≥140 mmHg, diastolic blood pressure ≥90 mmHg, previous medical history of diagnosed hypertension, or use of antihypertensive drugs [16].

The diagnosis of NAFLD was based on the abdominal ultrasound images. Experienced sonographers performed ultrasound at Tianjin Medical University General Hospital following a uniform standard protocol who were blinded to the patients' clinical information. NAFLD was diagnosed on the basis of high-level echoes from the hepatic parenchyma, differences in hepatorenal echo, echo penetration deep into the liver, and vascular blurring on ultrasonography [17].

### 2.4. Statistical Analyses

Normally distributed continuous variables were shown as mean ± standard deviation (SD) and compared between the groups using Student's *t*-test. Non-normally distributed continuous variables were expressed as medians with interquartile ranges (IQRs) and compared between the groups using the Mann–Whitney *U* test. Categorical variables were presented as numbers with frequencies and were compared between the groups using the chi-squared test. Binary logistic regression analyses were used to assess the contribution of the androgens to NAFLD after adjusting for confounding factors in four models: model 1 was adjusted for age and post-menopausal status (only for women); model 2 was adjusted for model 1 plus current smoking and current drinking; model 3 was adjusted for model 2 plus BMI, duration of T2DM, diastolic blood pressure, TC, TG, LDL/HDL cholesterol ratio, UA, and CRP; and model 4 was mutually adjusted for sex hormones in the basis of model 3. Factors that showed statistical significance in the univariate analysis, including age, post-menopausal status, BMI, duration of T2DM, diastolic blood pressure, TC, TG, LDL/HDL cholesterol ratio, UA, and CRP, or were considered clinically relevant, such as smoking and drinking, were included in the binary logistic regression analyses. The associations were shown as odds ratios (ORs) and 95% confidence intervals (CIs); *P* < 0.05 (two-tailed test) was considered statistically significant. SPSS for Windows (version 25.0; SPSS, Chicago, IL, USA) was used for all analyses.

## 3. Results

### 3.1. Characteristics of the Study Population with and without NAFLD

In the study population of 600 men, NAFLD was observed in 383 (63.8%). BMI, diastolic blood pressure, TC, TG, LDL-C, LDL/HDL cholesterol ratio, UA, AST, and ALT were higher in men with NAFLD than in those without NAFLD (all *P* < 0.05). Men diagnosed with NAFLD also had a higher prevalence of dyslipidemia (*P* = 0.002). However, the mean age, duration of T2DM, and HDL-C were lower in men with NAFLD than in those without NAFLD (all *P* < 0.001). As for TT and precursors of androgens, men diagnosed with NAFLD had higher DHEA and DHEAS levels but lower TT levels (all *P* < 0.001) (Table 1).

A total of 375 (67.6%) of the study population of 555 women were diagnosed with NAFLD. Similar to men, women with NAFLD had a higher BMI, systolic blood pressure, diastolic blood pressure, TC, TG, LDL-C, LDL/HDL cholesterol ratio, UA, CRP, AST, and ALT but lower mean age, duration of T2DM, and HDL-C than those without NAFLD (all *P* < 0.05). Women diagnosed with NAFLD had higher dyslipidemia but lower post-menopausal status rates (all *P* < 0.05). The level of DHEAS was higher in women with NAFLD (*P*=0.016, Table 2).

### 3.2. Prevalence of NAFLD by Tertiles of TT, Androstenedione, DHEA, and DHEAS

**Figure 2:**
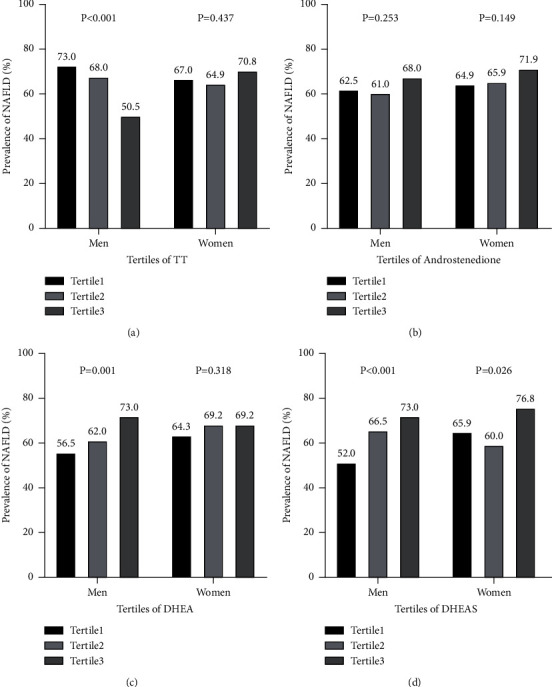
Prevalence of non-alcoholic fatty liver disease (NAFLD) by tertiles of serum androgens in male and female participants: (a) total testosterone; (b) androstenedione; (c) dehydroepiandrosterone (DHEA); (d) dehydroepiandrosterone sulfate (DHEAS). *P* values were analyzed using linear-by-linear association in chi-squared tests.

Figure 2shows the prevalence of NAFLD in men and women according to the tertiles of serum TT, androstenedione, DHEA, and DHEAS. The percentage of men with NAFLD significantly decreased with increasing tertiles of serum TT, with 73.0% in tertile1, 68.0% in tertile2, and 50.5% in tertile3 (*P* < 0.001). Furthermore, the prevalence of NAFLD in men gradually increased in line with increasing tertiles of DHEA and DHEAS, with 56.5% and 52.0% in tertile1, 62.0% and 66.5% in tertile2, and both 73.0% in tertile3, respectively (all *P* < 0.05). In women, we found that the percentage of NAFLD increased with increasing tertiles of DHEAS, with 65.9% in tertile1, 60.0% in tertile2, and 76.8% in tertile3 (*P*=0.026).

### 3.3. Associations of  TT, Androstenedione, DHEA, and DHEAS with the Risk of NAFLD

Table 3 presents the associations of TT, androstenedione, DHEA, and DHEAS with risk of NAFLD in men after adjusting for confounding variables in the multivariate logistic regression analysis. When only adjusted for age in model 1, the risk of NAFLD decreased with an increasing TT concentration. Even after adjusting for confounding variables in models 2, 3, and 4, this association remained robust. The fully adjusted OR (95% CI) for tertile3 vs tertile1 was 0.37 (0.17–0.77; *P*=0.024 for trend) in model 4. Similarly, when taken as a continuous variable, each SD increment in the TT level was significantly related to a 42% decrease in the odds of developing NAFLD in model 4 (OR, 0.58; 95% CI, 0.42–0.80; *P* < 0.05). No significant relationships were shown between increasing levels of androstenedione, DHEA, DHEAS, and the risk of NAFLD (all *P* > 0.05).

As shown in Table 4, women with T2DM showed no significant associations of increasing levels of TT, androstenedione, DHEA, and DHEAS, with the odds of NAFLD (all *P* > 0.05).

## 4. Discussion

In this cross-sectional study, we found that serum TT was significantly associated with NAFLD prevalence in men with T2DM. This finding remained significant even after adjusting for potential confounding factors, including age, current smoking, current drinking, BMI, duration of T2DM, diastolic blood pressure, TC, TG, LDL/HDL cholesterol ratio, UA, and CRP. In contrast, no statistically significant associations were found between TT levels and the risk of NAFLD in women with T2DM. Additionally, the participants showed no statistically significant relationships between increasing levels of androstenedione, DHEA, DHEAS, and NAFLD prevalence in men and women with T2DM.

Previous studies have shown that testosterone is involved in the incidence and progression of cardiovascular and metabolic diseases [18, 19]. NAFLD, which is causally related to metabolic syndrome [20], shares many potential risk factors for metabolic diseases. The inverse association between testosterone levels and the risk of NAFLD has been found in many studies among men. Nationally representative cross-sectional analyses in the United States (US) showed that the TT concentration was inversely related to the risk of NAFLD in men, independent of the age, obesity, and lifestyle [13, 21]. In addition, a cohort study by the multicenter NASH Clinical Research Network found that low testosterone levels were related to the development of NASH and fibrosis severity among men with NAFLD [12]. Moreover, in men with T2DM, an inverse association was found between serum TT and markers of liver fibrosis, including the FIB-4 index and 7S domain of type IV collagen [22]. Consistent with these studies, we concluded that, in men with T2DM, serum TT was significantly related to the risk of NAFLD after adjusting for potential confounding factors, including age, BMI, lifestyle, lipids, and uric acid. Therefore, an examination for NAFLD is essential for male patients with a low level of TT in clinical practice.

Mechanistically, the link between the liver and sex hormones is complex and bidirectional. One previous study showed that testosterone activated the androgen receptor/*β*-catenin interaction and subsequently translocated this complex to the nucleus, thus downregulating adipogenic transcription factors and inhibiting adipogenic differentiation [23]. In men, testosterone has been shown to inhibit lipoprotein lipase activity in the adipose tissue [24], which provides fat cells with free fatty acids for intracellular esterification by hydrolyzing circulating triglyceride-rich lipoproteins. For this reason, testosterone deficiency may contribute to visceral obesity and subsequent insulin resistance, which was found to upregulate glucose-6-phosphatase levels and increase hepatic gluconeogenesis [25]. In addition, the lack of hepatic androgen receptors decreased fatty acid beta-oxidation and increased de novo lipid synthesis, resulting in hepatic steatosis and insulin resistance [26]. On the other hand, high plasma levels of IL1*β*, which is a type of pro-inflammatory cytokine mainly produced by macrophages, may lead to decreased hepatic expression of sex hormone-binding globulin (SHBG) in patients with obesity [27]. Thus, fat deposition in the liver may affect the reproductive axis by regulating sex hormone metabolism through SHBG [28].

Significant benefits of testosterone treatment have been observed in animal and human research. In castrated male rats, testosterone replacement decreased micro and macrovesicular fat and apoptosis in hepatocytes, thus improving the NAFLD [29]. Similarly, long-term testosterone therapy produced a significant decline in the body weight, waist circumference, BMI, and lipid profile in men with hypogonadism [30–32]. In addition, testosterone therapy decreased the fatty liver index, gamma-glutamyl-transferase, and triglycerides, produced metabolically healthier preadipocytes, and improved mitochondrial functioning and lipid handling in hypogonadal men [33, 34]. Further prospective studies with larger sample sizes are needed to better understand the value of testosterone replacement for patients with metabolic diseases.

In women, the relationship between testosterone levels and the odds of NAFLD is controversial. A US study using data from 2011 to 2012 National Health and Nutrition Examination Survey reported that low TT levels were related to suspected NAFLD, diagnosed when serum ALT was greater than 19 IU/L, among post-menopausal women [21]. However, in the prospective Coronary Artery Risk Development in Young Adults study, high free testosterone was found to be related to NAFLD prevalence among middle-aged women, independent of traditional risk factors [35]. In middle-aged and elderly Chinese women living in the community, the calculated serum free testosterone and bioavailable testosterone levels were found to be positively associated with the risk of NAFLD, but TT was not significantly related to NAFLD [36]. In our study, we did not find a statistically significant relationship between TT and the odds of NAFLD among women with T2DM. In general, there are three different forms of testosterone in the circulation: free testosterone, testosterone bound to albumin, and testosterone bound to SHBG. Bioavailable testosterone forms, including free testosterone and albumin-bound testosterone, are readily available to cells. These different forms of testosterone could partly explain the different conclusions of the above studies, although further research is needed to clarify this issue.

As is well known, androstenedione, DHEA, and DHEAS, as precursors of androgens, are converted into testosterone and dihydrotestosterone in target organs and bind to androgen receptors to produce the effects of androgens. DHEA has been shown to increase insulin sensitivity [37] as well as predict all-cause and cardiovascular disease death [38]. The level of DHEA was lower in obese children aged 8.5 to 18.0 years with NAFLD than in those without liver disease [39]. An animal study also proved that DHEA can reduce tumor necrosis factor-*α*-induced hepatocyte apoptosis and, therefore, decrease liver injury in mice [40]. However, in the current study, no significant association was found between DHEA and the risk of NAFLD in either men or women with T2DM. These differences could possibly be explained by disparities in the study designs and populations.

DHEAS is the most abundant androgen precursor in circulation. In a retrospective study of women with polycystic ovary syndrome, the level of DHEAS was similar in patients with and without NAFLD when using ultrasound to diagnose this disease [41]. Nevertheless, other studies have reported that a low level of circulating DHEAS is histologically a crucial determinant of the severity of fibrosis in NAFLD [42, 43]. In the present cross-sectional study, we found no statistically significant relationship between DHEAS and the odds of NAFLD among individuals with T2DM. The differences in the criteria used to diagnose NAFLD as well as the specific populations included could be partly responsible for the conflicting results.

Androstenedione, which serves as a precursor in the biosynthesis of testosterone, is produced by the gonads and adrenal glands of both sexes. Few research studies have examined the relationship between androstenedione and the risk of NAFLD. Consistent with the results in children [44], this association was also not found to be statistically significant in patients with T2DM in this study.

This study has several limitations. First, we could not establish a causal relationship between androgens and NAFLD due to the cross-sectional nature of our study. Second, since the level of SHBG was not measured in our patients, we could not evaluate the role of free and bioavailable testosterone in the risk of developing NAFLD in patients with T2DM. Third, exercise and eating habits were not included in this study, which may be confounding factors in the development of NAFLD. Finally, the lack of histological evidence of NAFLD is another potential weakness of our study. Nevertheless, abdominal ultrasound is the preferred test for NAFLD in epidemiological studies because of the invasive nature of liver biopsy.

In conclusion, we found that serum TT was independently associated with the risk of NAFLD among men with T2DM in this cross-sectional study. In contrast, no statistically significant associations were found between precursors of androgens, including androstenedione, DHEA, and DHEAS, and the risk of NAFLD in men and women with T2DM. This study highlights the potential role of testosterone as a risk factor for NAFLD in patients with T2DM. Further longitudinal studies are needed to establish causal associations between androgens and the risk of NAFLD in the future.

## Figures and Tables

**Table 1:** Demographic characteristics and clinical parameters of patients with and without NAFLD (men, *n* = 600).

	Non-NAFLD	NAFLD	*P*
Participants, %	317 (36.2)	383 (63.8)	–
Age, years	62.15 ± 11.95	52.39 ± 14.98	<0.001
BMI, Kg/m^2^	24.29 ± 3.00	28.98 ± 5.29	<0.001
Current smoking, %	94 (43.3)	166 (44.4)	0.801
Current drinking^*∗*^, %	85 (39.2)	147 (39.3)	0.974
Insurance type, %			0.335
Urban workers	177 (81.6)	313 (81.7)	
Non-working urban residents	28 (12.9)	39 (10.2)	
Self-pay	12 (5.5)	31 (8.1)	
Dyslipidemia, %	156 (73.6)	320 (84.2)	0.002
Hypertension, %	127 (58.5)	233 (60.8)	0.579
Duration of T2DM, year	10.00 (6.00, 20.00)	5.00 (0.50, 13.00)	<0.001
Blood pressure, mmHg			
Systolic	134.95 ± 18.80	137.74 ± 17.85	0.072
Diastolic	80.82 ± 11.35	85.96 ± 11.74	<0.001
HbA1c, %	8.69 ± 2.29	8.78 ± 2.27	0.644
TC, mmol/L	4.50 ± 1.21	4.99 ± 1.97	0.001
TG, mmol/L	1.34 (0.95, 1.84)	2.00 (1.42, 2.96)	<0.001
HDL-C, mmol/L	1.11 ± 0.29	1.00 ± 0.23	<0.001
LDL-C, mmol/L	2.73 ± 0.96	3.00 ± 0.97	0.001
LDL/HDL cholesterol ratio	2.56 ± 0.95	3.10 ± 1.14	<0.001
UA, *μ*mol/L	343.13 ± 103.81	384.34 ± 107.49	<0.001
CRP, mg/dL	0.23 (0.16, 0.48)	0.27 (0.18, 0.48)	0.218
AST, u/l	16.00 (13.00, 20.00)	18.50 (15.00, 26.00)	<0.001
ALT, u/l	17.00 (12.00, 23.75)	24.00 (17.00, 38.00)	<0.001
TT, nmol/L	13.42 (10.00, 17.14)	11.36 (8.80, 14.27)	<0.001
Androstenedione, nmol/L	1.85 (1.41, 2.44)	1.94 (1.46, 2.52)	0.302
DHEA, nmol/L	6.53 (4.34, 10.12)	8.17 (5.37, 12.68)	<0.001
DHEAS, *μ*mol/L	3.02 (1.89, 4.91)	4.15 (2.58, 6.22)	<0.001

NAFLD, non-alcoholic fatty liver disease; BMI, body mass index; T2DM, type 2 diabetes mellitus; HbA1c, glycosylated hemoglobin; TC, total cholesterol; TG, triglycerides; HDL-C, high-density lipoprotein cholesterol; LDL-C, low-density lipoprotein cholesterol; UA, uric acid; CRP, C-reactive protein; AST, aspartate aminotransferase; ALT, alanine aminotransferase; TT, total testosterone; DHEA, dehydroepiandrosterone; DHEAS, dehydroepiandrosterone sulfate.^*∗*^Excess alcohol consumption (≥210 g/week for men, ≥140 g/week for women) was initially excluded.

**Table 2:** Demographic characteristics and clinical parameters of patients with and without NAFLD (women, *n* = 555).

	Non-NAFLD	NAFLD	*P*
Participants, %	180 (32.4)	375 (67.6)	–
Age, years	61.42 ± 13.32	54.40 ± 15.65	<0.001
BMI, Kg/m^2^	24.25 ± 3.68	29.52 ± 6.31	<0.001
Post-menopausal status, %	145 (80.6)	231 (61.6)	<0.001
Current smoking, %	7 (3.9)	9 (2.1)	0.523
Current drinking^*∗*^, %	4 (2.3)	13 (3.5)	0.423
Insurance type, %			0.802
Urban workers	144 (80.0)	297 (79.2)	
Non-working urban residents	27 (15.0)	54 (14.4)	
Self-pay	9 (5.0)	24 (6.4)	
Dyslipidemia, %	120 (69.0)	273 (73.6)	0.262
Hypertension, %	96 (53.3)	224 (59.7)	0.153
Duration of T2DM, year	10.00 (4.00, 20.00)	5.00 (0.40, 13.00)	<0.001
Blood pressure, mmHg			
Systolic	133.28 ± 18.55	136.70 ± 18.18	0.040
Diastolic	78.07 ± 10.49	82.33 ± 11.62	<0.001
HbA1c, %	8.33 ± 2.18	8.51 ± 2.00	0.363
TC, mmol/L	4.91 ± 1.12	5.18 ± 1.48	0.039
TG, mmol/L	1.43 (1.03, 1.90)	1.89 (1.45, 2.70)	<0.001
HDL-C, mmol/L	1.22 ± 0.33	1.09 ± 0.24	<0.001
LDL-C, mmol/L	2.89 ± 0.93	3.10 ± 1.01	0.018
LDL/HDL cholesterol ratio	2.52 ± 0.98	2.95 ± 1.15	<0.001
UA, *μ*mol/L	305.23 ± 103.28	350.08 ± 103.40	<0.001
CRP, mg/dL	0.23 (0.15, 0.39)	0.36 (0.22, 0.58)	<0.001
AST, u/l	16.00 (13.00, 20.00)	20.00 (15.00.29.00)	<0.001
ALT, u/l	16.00 (12.00, 23.00)	24.00 (16.00, 40.00)	<0.001
TT, nmol/L	0.45 (0.32, 0.75)	0.49 (0.32, 0.74)	0.283
Androstenedione, nmol/L	1.63 (1.16, 2.39)	1.78 (1.13, 2.72)	0.084
DHEA, nmol/L	7.67 (4.59, 12.39)	8.69 (4.80, 12.76)	0.395
DHEAS, *μ*mol/L	2.17 (1.30, 3.23)	2.48 (1.51, 4.16)	0.016

NAFLD, non-alcoholic fatty liver disease; BMI, body mass index; T2DM, type 2 diabetes mellitus; HbA1c, glycosylated hemoglobin; TC, total cholesterol; TG, triglycerides; HDL-C, high-density lipoprotein cholesterol; LDL-C, low-density lipoprotein cholesterol; UA, uric acid; CRP, C-reactive protein; AST, aspartate aminotransferase; ALT, alanine aminotransferase; TT, total testosterone; DHEA, dehydroepiandrosterone; DHEAS, dehydroepiandrosterone sulfate.^*∗*^Excess alcohol consumption (≥210 g/week for men, ≥140 g/week for women) was initially excluded.

**Table 3:** Odds ratios of NAFLD among men by different types of androgens.

	Odds ratios (95% CI)			
	Model 1	Model 2	Model 3	Model 4
TT				
Tertile1	1	1	1	1
Tertile2	0.76 (0.48, 1.20)	0.77 (0.48, 1.21)	0.70 (0.35, 1.40)	0.68 (0.33, 1.38)
Tertile3	0.36 (0.23, 0.57)	0.37 (0.24, 0.58)	0.38 (0.19, 0.77)	0.37 (0.17, 0.77)
P for trend	<0.001	<0.001	0.018	0.024
Per SD increment	0.61 (0.50, 0.73)	0.61 (0.51, 0.74)	0.58 (0.42, 0.79)	0.58 (0.42, 0.80)

Androstenedione				
Tertile1	1	1	1	1
Tertile2	0.72 (0.47, 1.10)	0.74 (0.48, 1.14)	0.74 (0.40, 1.38)	0.76 (0.38, 1.54)
Tertile3	0.89 (0.57, 1.39)	0.93 (0.59, 1.45)	0.89 (0.46, 1.72)	0.97 (0.43, 2.18)
P for trend	0.307	0.349	0.634	0.683
Per SD increment	0.93 (0.77, 1.11)	0.93 (0.78, 1.12)	0.90 (0.68, 1.18)	0.91 (0.63, 1.30)

DHEA				
Tertile1	1	1	1	1
Tertile2	0.84 (0.54, 1.28)	0.87 (0.56, 1.34)	0.92 (0.50, 1.72)	1.13 (0.56, 2.29)
Tertile3	0.83 (0.50, 1.36)	0.85 (0.51, 1.40)	1.11 (0.53, 2.33)	1.57 (0.57, 4.29)
P for trend	0.664	0.757	0.886	0.671
Per SD increment	0.98 (0.78, 1.24)	0.97 (0.77, 1.23)	1.03 (0.73, 1.47)	1.22 (0.71, 2.09)

DHEAS				
Tertile1	1	1	1	1
Tertile2	1.36 (0.89, 2.07)	1.37 (0.89, 2.09)	1.73 (0.93, 3.21)	1.48 (0.77, 2.88)
Tertile3	1.14 (0.70, 1.84)	1.14 (0.70, 1.85)	1.08 (0.51, 2.27)	0.81 (0.33, 2.00)
P for trend	0.370	0.352	0.179	0.239
Per SD increment	1.07 (0.86, 1.33)	1.06 (0.85, 1.32)	1.13 (0.79, 1.61)	0.99 (0.65, 1.52)

Model 1: adjusted for age. Model 2: adjusted for model 1 + current smoking and current drinking. Model 3: adjusted for model 2 + BMI, duration of T2DM, diastolic blood pressure, TC, TG, LDL/HDL cholesterol ratio, UA, and CRP. Model 4: adjusted for model 3, but mutually for hormones. NAFLD, non-alcoholic fatty liver disease; CI, confidence interval; SD, standard deviation; BMI, body mass index; TC, total cholesterol; TG, triglycerides; HDL-C, high-density lipoprotein cholesterol; LDL-C, low-density lipoprotein cholesterol; UA, uric acid; CRP, C-reactive protein; TT, total testosterone; DHEA, dehydroepiandrosterone; DHEAS, dehydroepiandrosterone sulfate.

**Table 4:** Odds ratios of NAFLD among women by different types of androgens.

	Odds ratios (95% CI)			
	Model 1	Model 2	Model 3	Model 4
TT				
Tertile1	1	1	1	1
Tertile2	0.87 (0.56, 1.35)	0.88 (0.56, 1.36)	0.70 (0.37, 1.31)	0.75 (0.39, 1.45)
Tertile3	0.96 (0.61, 1.52)	0.96 (0.60, 1.52)	0.84 (0.44, 1.61)	0.92 (0.44, 1.92)
P for trend	0.807	0.832	0.532	0.664
Per SD increment	0.91 (0.75, 1.09)	0.91 (0.76, 1.10)	0.81 (0.50, 1.31)	0.74 (0.41, 1.30)

Androstenedione				
Tertile1	1	1	1	1
Tertile2	0.93 (0.60, 1.44)	0.89 (0.58, 1.39)	0.79 (0.43, 1.46)	0.88 (0.41, 1.87)
Tertile3	0.82 (0.50, 1.34)	0.82 (0.50, 1.35)	0.79 (0.40, 1.59)	0.91 (0.34, 2.45)
P for trend	0.732	0.731	0.709	0.941
Per SD increment	1.00 (0.80, 1.26)	1.01 (0.80, 1.26)	1.01 (0.74, 1.40)	1.20 (0.77, 1.88)

DHEA				
Tertile1	1	1	1	1
Tertile2	1.04 (0.67, 1.63)	1.06 (0.67, 1.66)	0.77 (0.41, 1.46)	1.16 (0.51, 2.66)
Tertile3	0.72 (0.44, 1.17)	0.70 (0.43, 1.14)	0.84 (0.43, 1.64)	1.41 (0.52, 3.84)
P for trend	0.272	0.212	0.721	0.791
Per SD increment	0.86 (0.71, 1.05)	0.85 (0.70, 1.04)	0.90 (0.64, 1.27)	0.96 (0.59, 1.55)

DHEAS				
Tertile1	1	1	1	1
Tertile2	0.67 (0.43, 1.03)	0.66 (0.43, 1.03)	0.56 (0.30, 1.03)	0.52 (0.26, 1.05)
Tertile3	1.10 (0.66, 1.80)	1.06 (0.64, 1.76)	0.58 (0.28, 1.20)	0.52 (0.21, 1.25)
P for trend	0.070	0.082	0.139	0.171
Per SD increment	1.10 (0.88, 1.37)	1.07 (0.86, 1.34)	0.84 (0.61, 1.16)	0.86 (0.57, 1.28)

Model 1: adjusted for age and post-menopausal status. Model 2: adjusted for model 1 + current smoking and current drinking. Model 3: adjusted for model 2 + BMI, duration of T2DM, diastolic blood pressure, TC, TG, LDL/HDL cholesterol ratio, UA, and CRP. Model 4: adjusted for model 3, but mutually for hormones. NAFLD, non-alcoholic fatty liver disease; CI, confidence interval; SD, standard deviation; BMI, body mass index; TC, total cholesterol; TG, triglycerides; HDL-C, high-density lipoprotein cholesterol; LDL-C, low-density lipoprotein cholesterol; UA, uric acid; CRP, C-reactive protein; TT, total testosterone; DHEA, dehydroepiandrosterone; DHEAS, dehydroepiandrosterone sulfate.

## Data Availability

The dataset generated and analyzed during the current study is available from the corresponding author upon reasonable request.

## References

[1] Younossi Z. M., Koenig A. B., Abdelatif D., Fazel Y., Henry L., Wymer M. (2016). Global epidemiology of nonalcoholic fatty liver disease-Meta-analytic assessment of prevalence, incidence, and outcomes. *Hepatology (Baltimore, Md)*.

[2] Torres D. M., Williams C. D., Harrison S. A. (2012). Features, diagnosis, and treatment of nonalcoholic fatty liver disease. *Clinical Gastroenterology and Hepatology*.

[3] Xiao J., Wang F., Wong N. K. (2019). Global liver disease burdens and research trends: analysis from a Chinese perspective. *Journal of Hepatology*.

[4] Younossi Z. M., Otgonsuren M., Venkatesan C., Mishra A. (2013). In patients with non-alcoholic fatty liver disease, metabolically abnormal individuals are at a higher risk for mortality while metabolically normal individuals are not. *Metabolism*.

[5] Jao H. F., Wung C. H., Yu H. C. (2021). Sex difference in the associations among obesity-related indices with metabolic syndrome in patients with type 2 diabetes mellitus. *International Journal of Medical Sciences*.

[6] Pan J. J., Fallon M. B. (2014). Gender and racial differences in nonalcoholic fatty liver disease. *World Journal of Hepatology*.

[7] Tobari M., Hashimoto E. (2020). Characteristic features of nonalcoholic fatty liver disease in Japan with a focus on the roles of age, sex and body mass index. *Gut and Liver*.

[8] Lonardo A., Nascimbeni F., Ballestri S. (2019). Sex differences in nonalcoholic fatty liver disease: state of the art and identification of research gaps. *Hepatology (Baltimore, Md)*.

[9] Powell E. E., Wong V. W. S., Rinella M. (2021). Non-alcoholic fatty liver disease. *The Lancet*.

[10] Medina J., Fernández-Salazar L. I., García-Buey L., Moreno-Otero R. (2004). Approach to the pathogenesis and treatment of nonalcoholic steatohepatitis. *Diabetes Care*.

[11] Van de Velde F., Bekaert M., Hoorens A. (2020). Histologically proven hepatic steatosis associates with lower testosterone levels in men with obesity. *Asian Journal of Andrology*.

[12] Sarkar M., Yates K., Suzuki A. (2021). Low testosterone is associated with nonalcoholic steatohepatitis and fibrosis severity in men. *Clinical Gastroenterology and Hepatology*.

[13] Phan H., Richard A., Lazo M. (2021). The association of sex steroid hormone concentrations with non-alcoholic fatty liver disease and liver enzymes in US men. *Liver International*.

[14] branch of the Chinese Medical Association D. (2021). Guidelines for the prevention and treatment of type 2 diabetes in China (2020 edition). *Chin J Endocrinol Metab*.

[15] (2016). The joint committee of Chinese adult dyslipidemia prevention guide. “Guidelines on prevention and treatment of dyslipidemia in Chinese adults (2016 edition)”. *Chin J Cardiol*.

[16] (2019). The joint committee of Chinese hypertension prevention guide. “Guidelines on prevention and treatment of hypertension in China (2018 edition)”. *Chin J Cardiovasc*.

[17] (2010). The Chinese national workshop on fatty liver and alcoholic liver disease for the Chinese liver disease association. “Guidelines for the diagnosis and management of nonalcoholic fatty liver disease: update 2010”. *Chin J Hepatol*.

[18] Pastuszak A. W., Kohn T. P., Estis J., Lipshultz L. I. (2017). Low plasma testosterone is associated with elevated cardiovascular disease biomarkers. *The Journal of Sexual Medicine*.

[19] Bianchi V. E., Locatelli V. (2018). Testosterone a key factor in gender related metabolic syndrome. *Obesity Reviews*.

[20] Yki-Järvinen H. (2014). Non-alcoholic fatty liver disease as a cause and a consequence of metabolic syndrome. *The Lancet Diabetes & Endocrinology*.

[21] Yim J. Y., Kim J., Kim D., Ahmed A. (2018). Serum testosterone and non-alcoholic fatty liver disease in men and women in the US. *Liver International*.

[22] Miyauchi S., Miyake T., Miyazaki M. (2017). Free testosterone concentration is inversely associated with markers of liver fibrosis in men with type 2 diabetes mellitus. *Endocrine Journal*.

[23] Singh R., Artaza J. N., Taylor W. E. (2006). Testosterone inhibits adipogenic differentiation in 3T3-L1 cells: nuclear translocation of androgen receptor complex with beta-catenin and T-cell factor 4 may bypass canonical Wnt signaling to down-regulate adipogenic transcription factors. *Endocrinology*.

[24] Ramirez M. E., McMurry M. P., Wiebke G. A. (1997). Evidence for sex steroid inhibition of lipoprotein lipase in men: comparison of abdominal and femoral adipose tissue. *Metabolism*.

[25] Aoki A., Fujitani K., Takagi K., Kimura T., Nagase H., Nakanishi T. (2016). Male hypogonadism causes obesity associated with impairment of hepatic gluconeogenesis in mice. *Biological & Pharmaceutical Bulletin*.

[26] Lin H. Y., Yu I. C., Wang R. S. (2008). Increased hepatic steatosis and insulin resistance in mice lacking hepatic androgen receptor. *Hepatology (Baltimore, Md)*.

[27] Simó R., Barbosa-Desongles A., Hernandez C., Selva D. M. (2012). IL1*β* down-regulation of sex hormone-binding globulin production by decreasing HNF-4*α* via MEK-1/2 and JNK MAPK pathways. *Molecular Endocrinology (Baltimore, Md)*.

[28] Hammond GL. (1995). Potential functions of plasma steroid-binding proteins. *Trends in Endocrinology & Metabolism*.

[29] Nikolaenko L., Jia Y., Wang C. (2014). Testosterone replacement ameliorates nonalcoholic fatty liver disease in castrated male rats. *Endocrinology*.

[30] Saad F., Doros G., Haider K. S., Haider A. (2005). Differential effects of 11 years of long-term injectable testosterone undecanoate therapy on anthropometric and metabolic parameters in hypogonadal men with normal weight, overweight and obesity in comparison with untreated controls: real-world data from a controlled registry study. *International Journal of Obesity*.

[31] Saad F., Yassin A., Doros G., Haider A. (2005). Effects of long-term treatment with testosterone on weight and waist size in 411 hypogonadal men with obesity classes I-III: observational data from two registry studies. *International Journal of Obesity*.

[32] Saad F., Haider A., Doros G., Traish A. (2013). Long-term treatment of hypogonadal men with testosterone produces substantial and sustained weight loss. *Obesity (Silver Spring, Md)*.

[33] Yassin A. A., Alwani M., Talib R. (2020). Long-term testosterone therapy improves liver parameters and steatosis in hypogonadal men: a prospective controlled registry study. *The Aging Male*.

[34] Maseroli E., Comeglio P., Corno C. (2021). Testosterone treatment is associated with reduced adipose tissue dysfunction and nonalcoholic fatty liver disease in obese hypogonadal men. *Journal of Endocrinological Investigation*.

[35] Sarkar M., Wellons M., Cedars M. I. (2017). Testosterone levels in pre-menopausal women are associated with nonalcoholic fatty liver disease in midlife. *American Journal of Gastroenterology*.

[36] Wang X., Li Q., Pang J. (2021). Associations between serum total, free and bioavailable testosterone and non-alcoholic fatty liver disease in community-dwelling middle-aged and elderly women. *Diabetes & Metabolism*.

[37] Dhatariya K., Bigelow M. L., Nair K. S. (2005). Effect of dehydroepiandrosterone replacement on insulin sensitivity and lipids in hypoadrenal women. *Diabetes*.

[38] Ohlsson C., Labrie F., Barrett-Connor E. (2010). Low serum levels of dehydroepiandrosterone sulfate predict all-cause and cardiovascular mortality in elderly Swedish men. *The Journal of Clinical Endocrinology & Metabolism*.

[39] Gawlik A., Shmoish M., Hartmann M. F. (2019). Steroid metabolomic signature of liver disease in nonsyndromic childhood obesity. *Endocrine Connections*.

[40] Yoneda M., Wada K., Katayama K. (2004). A novel therapy for acute hepatitis utilizing dehydroepiandrosterone in the murine model of hepatitis. *Biochemical Pharmacology*.

[41] Kauffman R. P., Baker T. E., Baker V., Kauffman M. M., Castracane V. D. (2010). Endocrine factors associated with non-alcoholic fatty liver disease in women with polycystic ovary syndrome: do androgens play a role?. *Gynecological Endocrinology*.

[42] Sumida Y., Yonei Y., Kanemasa K. (2010). Lower circulating levels of dehydroepiandrosterone, independent of insulin resistance, is an important determinant of severity of non-alcoholic steatohepatitis in Japanese patients. *Hepatology Research*.

[43] Tokushige K., Hashimoto E., Kodama K. (2013). Serum metabolomic profile and potential biomarkers for severity of fibrosis in nonalcoholic fatty liver disease. *Journal of Gastroenterology*.

[44] Mueller N. T., Liu T., Mitchel E. B. (2020). Sex hormone relations to histologic severity of pediatric nonalcoholic fatty liver disease. *The Journal of Clinical Endocrinology & Metabolism*.

